# A Novel Telescopic Access Sheath Method to Manage Encrusted or Knotted Retained Ureteral Stents

**DOI:** 10.1089/end.2021.0942

**Published:** 2022-07-05

**Authors:** Dinesh K. Agarwal

**Affiliations:** ^1^ Department of Urology, Royal Melbourne Hospital, Parkville, VIC, Australia.; ^2^ Department of Urology, Western Health, Footscray, VIC, Australia.; ^3^ Department of Urology, Werribee Mercy Hospital, Werribee, VIC, Australia.

**Keywords:** ureter, stents, encrustation, knotted stents, ureteral stents

## Abstract

**Purpose:**

Encrusted and knotted stents may cause serious urologic complications. This study aimed to develop a novel and minimally invasive technique to manage encrusted or knotted retained ureteral stents.

**Materials and Methods:**

This technique was used in nine patients with retained stents. Through rigid cystoscopy, the stents were pulled out of the urinary meatus. The access sheath was modified by cutting the distal end of its obturator. The modified access sheath was advanced over the retained stents in a telescopic manner to remove the encrusted and/or knotted stents.

**Results:**

Six patients had encrustations, two had knot formation, and one had both encrustation and knot formation. The encrustations were peeled off in the process. The knots were either undone or pulled through the lumen of the access sheath. The retained stents were removed intact from all patients without any complications.

**Conclusion:**

The access sheath method described in this article provides a simple alternative for the removal of encrusted or knotted retained stents. However, this technique requires further validation to establish its safety and efficacy.

## Introduction

Ureteral stents, also known as double-J-stents, have been widely used in urology practice since their first description in 1967.^1^ They are commonly used to relieve obstruction of the upper urinary tract. They are also used as an adjunct for various urologic procedures. However, ureteral stents may be associated with complications such as pain, hematuria, infection, encrustation, and, rarely, knot formation in the upper curl within the renal pelvis.^2,3^

Encrustations can occur in up to 13% of cases, especially if stents are left for longer periods or forgotten.^4^ There are no formal guidelines for the maximum safe indwelling time for ureteral stents with a minimal risk for developing encrustations. The mechanism of encrustations is complex and multifactorial; it includes dwell time, stent composition, patient-specific factors, biofilm formation, and mineral deposition.^5–9^ Encrusted retained ureteral stents are a critical problem if they cannot be removed by a simple cystoscopic procedure.

Depending upon the site and degree of encrustation, these cases may require additional procedures.^10–18^ Similarly, the management of retained knotted stents is a clinical dilemma and may require further endoscopic or percutaneous procedures.^3,19–26^ These additional procedures to remove the retained stents may lead to high morbidity in patients. Up to 16% of endourology lawsuits in the United States are related to retained stents.^27^ Thus, there is a requirement for simpler and noninvasive techniques that could remove the retained stents without causing any complications. This article aims to describe a simple and minimally invasive method, using a modified access sheath, to remove the encrusted and/or knotted retained stents.

## Materials and Methods

This project received approval from the Royal Melbourne Hospital Human Research Ethics Committee.

### Initial preparation

**FIG. 1.**
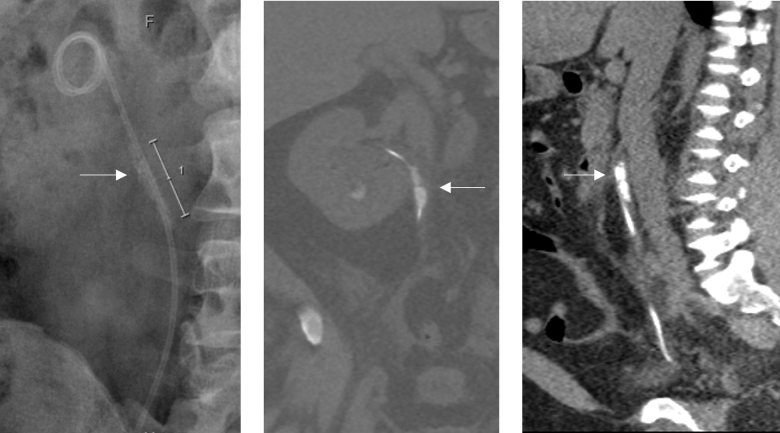
KUB radiograph and CT KUB imaging showing encrustations (indicated by *arrow*) in the upper part of the ureteral stent. KUB, kidney, ureter, and bladder.

**FIG. 2.**
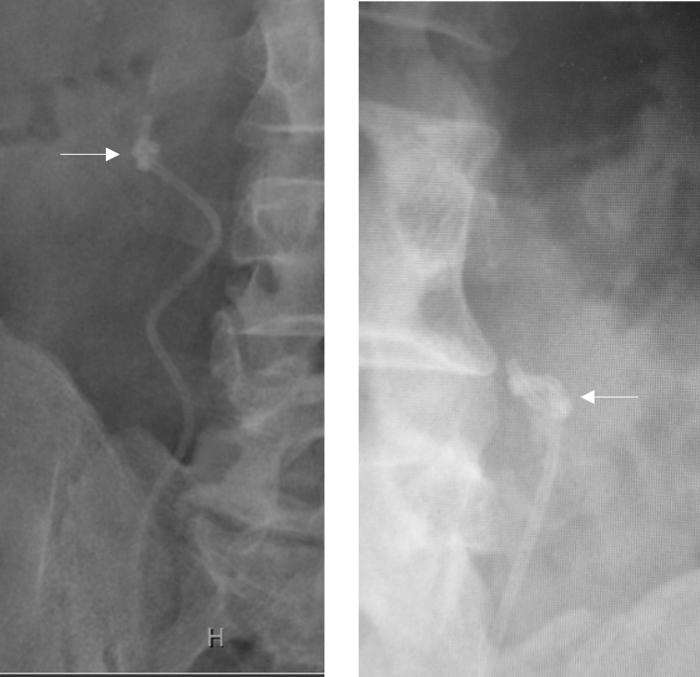
KUB X-ray showing a knot (indicated by *arrow*) in the upper curl of the ureteral stent.

The telescopic access sheath method was used in nine patients with encrusted (Fig. 1) and/or knotted retained stents (Fig. 2). Rigid cystoscopy was performed in the lithotomy position under general anesthesia. The lower curl of the ureteral stent was observed and the distal end of the ureteral stent was grasped and gently pulled out of the urinary meatus. The end of the stent was secured to avoid retraction into the urethra.

We used ureteral access sheaths from Applied Medical, US (12F/14F); Boston Scientific, US (13F/15F); and Cook Medical, US (14F/16F). Ureteral access sheaths are commonly used to facilitate the passage of flexible ureteroscopes into the ureter and the upper collecting system. They have two components: an inner tapered obturator and an outer sheath. The lumen of the obturator is not uniform. It is wider proximally and tapered at the tip to accommodate a 0.038 inch (0.97 mm) diameter guidewire. The proximal lumens of the obturators of Applied Medical's (12F/14F), Cook Medical's (14F/16F), and Boston Scientific's (13F/15F) sheaths can accommodate 6F, 7F, and 8F ureteral catheters, respectively.

The tapered end of the obturator was cut with a surgical blade to expose its dilated lumen and to accommodate the distal end of the ureteral stent. This obturator was used with the outer sheath to remove the retained stent. In one patient with an encrusted stent, the obturator was used alone. The technique for removing the retained stents is described in the following sections.

### Technique for removing the encrusted stents

**FIG. 3.**
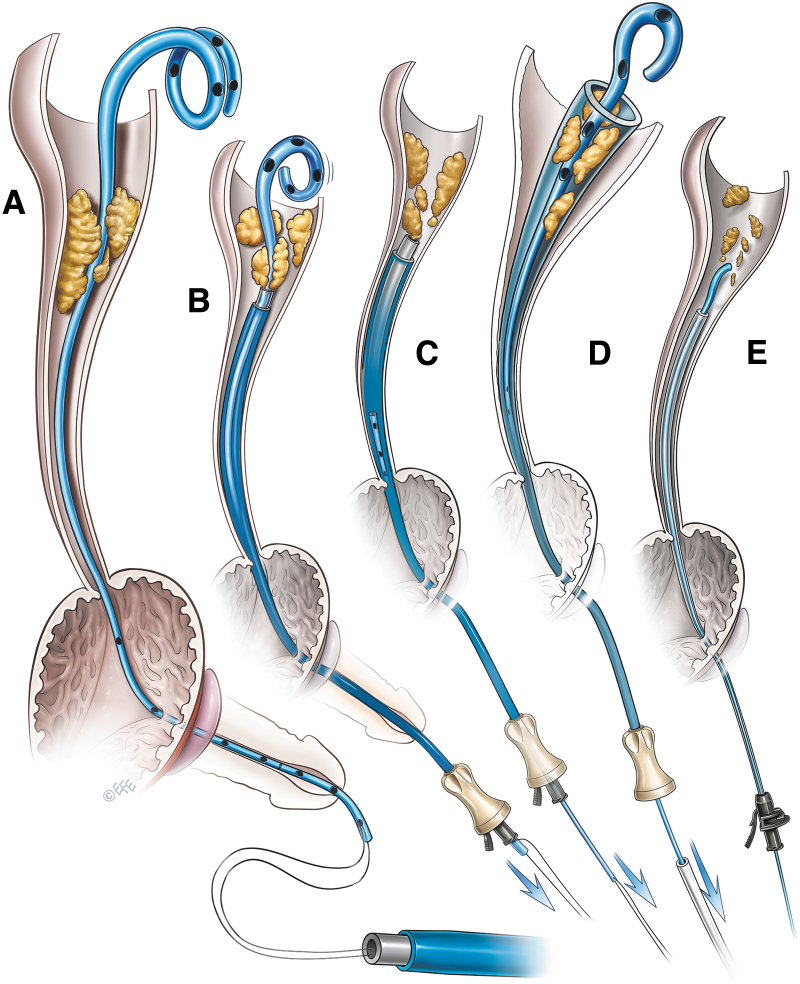
Telescopic access sheath method of removing an encrusted ureteral stent. **(A)** The lower end of the ureteral stent is pulled out of the urinary meatus and secured with a 2–0 Prolene suture. Both ends of the suture are fed through the lumen of the obturator to retrieve them from the proximal end. **(B)** Access sheath with an obturator is gently telescoped over the ureteral stent up to the site of the encrustations. **(C)** Encrustations are peeled off and the ureteral stent is removed. **(D)** The ureteral stent with mild encrustations is removed by pulling it inside the access sheath after removing the obturator. **(E)** The obturator alone is telescoped over the ureteral stent to peel off the encrustations.

The technique used to remove the encrusted stent is shown in Figure 3. The lower end of the ureteral stent was secured with a 2–0 Prolene suture (SH, 26 mm ½ circle taper needle; Ethicon 8533, US) by passing it through the lowest side hole and the luminal end. This suture was double-armed and 122 cm long with one needle cut before use. After securing the lower end of the stent, another needle was cut. Both the free ends of the suture were fed into the lumen of the obturator of the access sheath and retrieved from the proximal end (Fig. 3A).

The suture was long enough to be retrieved from the proximal end of the access sheaths of varying lengths. The obturator along with the outer sheath was then gently advanced into the ureter over the ureteral stent up to the site of encrustations. The process was done in a telescopic manner under fluoroscopic guidance. During the process, the Prolene suture was held tight with mild tension (Fig. 3B). As the access sheath progressively advanced, the lower end of the stent was retrieved from the proximal end of the sheath when encrustations involved the upper end of the stent.

**FIG. 4.**
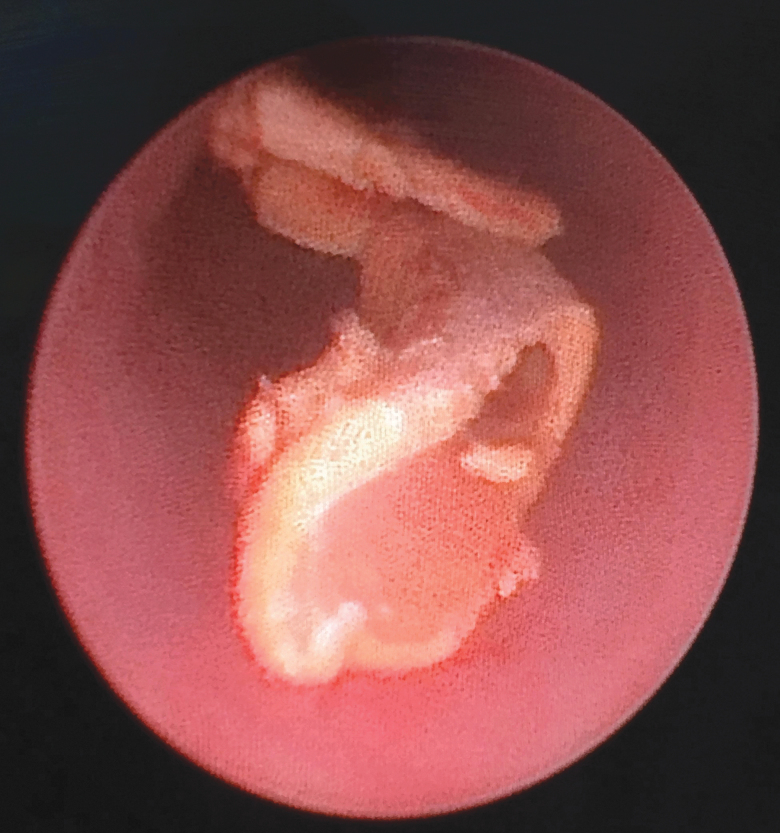
Ureteroscopic view of a peeled off stone fragment.

To peel off the encrustations from the stent, gentle pressure was applied repeatedly with the access sheath assembly by holding the Prolene suture or the lower end of the stent under mild tension (Fig. 3C). Patience is required during this stage of the procedure, as it may take a few minutes to clear the encrustations from the stent. Excessive pressure should be avoided, as it may break the stent. In one patient with mild encrustations at the upper end of the ureteral stent, the encrusted stent was gently pulled inside the access sheath after removing the obturator, and the stent was removed (Fig. 3D). In another patient, we used a refashioned obturator without an outer access sheath to peel off the encrustations and remove the ureteral stent (Fig. 3E). Ureteroscopy was performed to treat the residual stone fragments (Fig. 4). A new ureteral stent was placed, at the end of the procedure, in all the patients. This stent was removed after 1–2 weeks.

### Technique for removing the knotted stents

In patients with knotted stents, we tried to undo the knot using a stiff guidewire, but this maneuver failed in all cases.

**FIG. 5.**
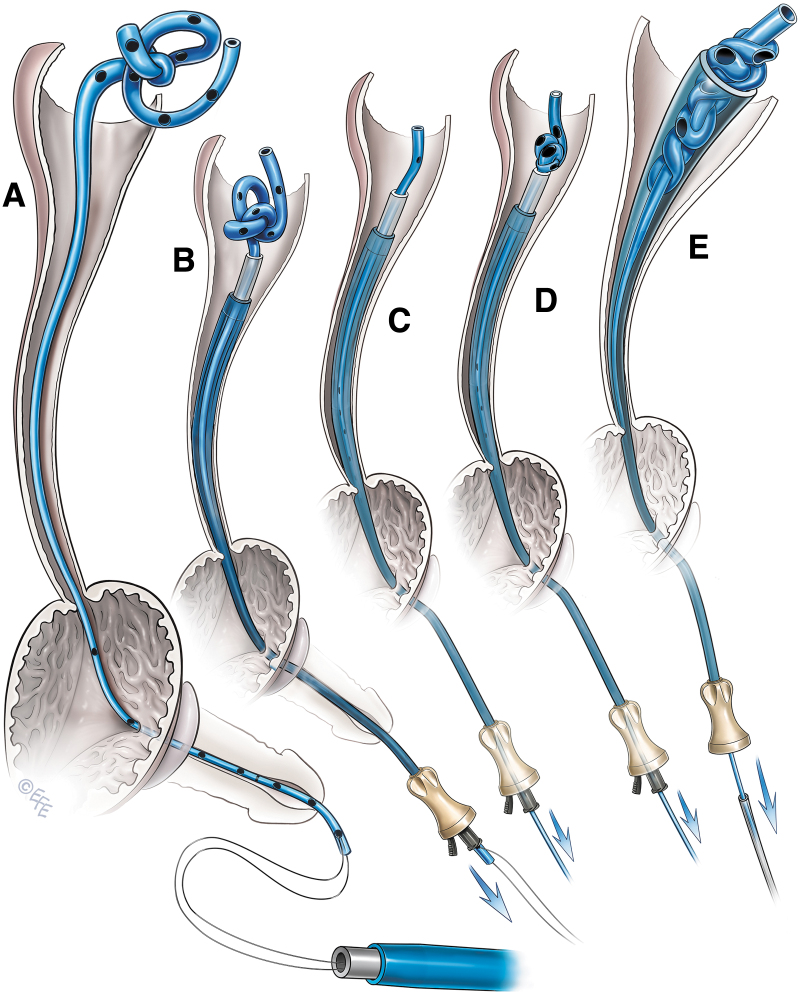
Telescopic access sheath method of removing a knotted ureteral stent. **(A)** The lower end of the ureteral stent is pulled out of the urinary meatus and secured with a 2–0 Prolene suture. Both ends of the suture are fed through the lumen of the obturator to retrieve them from the proximal end. **(B)** Access sheath with the obturator is gently telescoped over the ureteral stent up to the knot. **(C)** The knot is undone by gently pulling the ureteral stent from outside. **(D)** If the knot cannot be undone, the ureteral stent is gently pulled to decrease the size of the knot. **(E)** The obturator is removed, and the knot is gently pulled inside the access sheath to remove the stent.

**FIG. 6.**
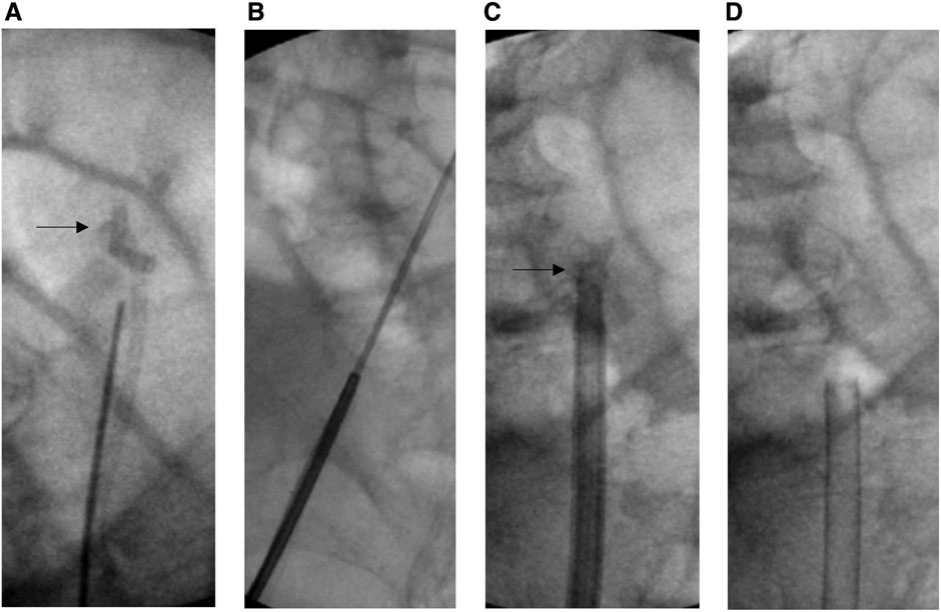
Fluoroscopic images of knotted stent removal. **(A)** A knot in the upper curl of the ureteral stent (indicated by *arrow*). **(B)** Modified access sheath assembly being telescoped over the ureteral stent. **(C)** The knot (indicated by *arrow*) is pulled inside the access sheath after removing the obturator. **(D)** The knotted stent is pulled out through the lumen of the access sheath.

Details of the technical steps for removing the knotted stent are shown in Figure 5. In two patients with a knot in the stent, the obturator along with the outer sheath assembly was used. The lower end of the ureteral stent was passed through the lumen of the access sheath assembly as described earlier (Fig. 5A). Under fluoroscopic guidance, the access sheath assembly was gently advanced over the ureteral stent in a telescopic manner up to the knot (Figs. 5B and 6). At this stage, the lower end of the stent emerged from the lower end of the access sheath.

Gentle pressure was applied by pulling down the lower end of the stent. The knot was easily undone in one patient and the stent was removed (Fig. 5C). In another patient, the knot could not be undone. Hence, we attempted to tighten the knot and decrease its size by gently pulling the lower end of the stent (Fig. 5D). Excessive pulling pressure was avoided to prevent inadvertent stent breakage. At this stage, we removed the obturator of the access sheath, pulled the knot inside the access sheath, and pulled the stent out (Figs. 5E and 6).

In a patient with both encrustation and a knot in the stent, we also used the obturator and an outer sheath assembly. The access sheath assembly was advanced over the ureteral stent, as described earlier, up to the site of encrustation. Encrustations were cleared by gentle and repetitive pushing of the access sheath over the stent under fluoroscopic guidance, as described earlier. Once the encrustations were cleared, the access sheath assembly was gently advanced to the site of the knot. The knot was tightened to reduce its size. Then, the obturator was removed and the knotted stent was pulled out through the access sheath.

## Results

The demographics of the nine patients with retained stents in this study are outlined in Table 1. Among them, seven patients were men and two were women. Their ages ranged from 36 to 77 years. In all patients, stents were placed after ureteroscopy for stone management. These stents could not be removed using a flexible cystoscope under local anesthesia. Plain kidney, ureter, and bladder (KUB) radiography and/or plain CT KUB showed encrustations of the ureteral stent in six patients, knots in the upper curl of the ureteral stent in two patients, and both knots and encrustations in one patient. All ureteral stents were either from Boston Scientific or Cook Medical and were ≤5F in size.

**Table 1.** Demographics of the Patients

Age	Gender	Site	Pathology report of retained stent	Location	Duration of stent (weeks)
77	M	Right	Encrustations	Renal pelvis	16
36	M	Right	Encrustations	Upper ureter	8
60	F	Left	Encrustations	Renal pelvis	68
48	M	Right	Encrustations	Upper ureter	45
52	F	Left	Encrustations	Renal pelvis	30
58	M	Left	Encrustations	Upper ureter	36
77	M	Left	Knot	Renal pelvis	4
44	M	Left	Knot	Renal pelvis	3
65	M	Right	Knot	Renal pelvis	9
			Encrustations	Upper ureter	

Using the telescopic modified access sheath method, the retained stents were removed in all patients, without any complications. No suture breakage occurred during the advancement of the access sheath assembly over the ureteral stents. Encrustations were peeled off and the stents were removed intact in all the patients. All stone fragments were treated by ureteropyeloscopy using a holmium laser. Similarly, all the knotted stents were removed intact. Retrograde pyelography findings were normal in all these patients with no evidence of ureteral injury. All patients were discharged on the same day.

## Discussion

The management of encrusted and/or knotted retained ureteral stents poses a surgical challenge for urologists. In this study, we removed the encrusted and/or knotted stents using a novel technique. Encrustation of ureteral stents is a clinical problem because of their widespread use in various urologic procedures. The treatment of the encrusted ureteral stent may depend on the site and degree of encrustation. Several grading systems exist to define the extent of encrustations and predict the surgical complexity of stent removal.^9,28–30^ Patients in this study mostly had a low grade of encrustation involving the upper part of the stent. Patients with encrustation may require various invasive procedures such as retrograde ureteroscopic manipulation, intracorporeal lithotripsy, holmium laser stent cutting, extracorporeal lithotripsy, percutaneous nephrolithotomy, and open surgery, either alone or in combination.^10–17^ Some patients may even require multiple sessions to clear the encrustations.^18^

We, for the first time, have described the telescopic access sheath method to remove the retained encrusted stents. This method is comparatively simpler and less invasive than other methods described in the literature. This procedure was effective in all patients without any complications. As none of the patients had a high grade of encrustations, this technique may be suitable for patients with a lower degree of encrustation. In one patient with encrustations, we used an obturator without an outer access sheath. However, obturators without an outer sheath have a greater tendency to buckle in the ureter, posing a potential risk for ureteral injury. Thus, the use of an obturator with an outer sheath is recommended to avoid buckling and potential ureteral injury.

Although rare, another issue with retained ureteral stents is knot formation in the upper curl. There are a few reports of knotted ureteral stents in the medical literature; however these are either mostly isolated case reports or small case series.^3,19–26^ It is speculated that knot formation may occur due to the excessive length of the coils.^25,26^ A variety of techniques have been described to remove the knotted stent. Among them, gentle traction and unknotting of the stent by passing a super-stiff guidewire may represent a minimally invasive technique.^3,20^ However, this technique has a potential risk for ureteral avulsion. In our study, unknotting with a super-stiff guidewire was ineffective in the patients.

Retrograde ureteroscopy and holmium laser fragmentation of the knot has also been reported to have effective outcomes.^22,24,26^ However, passing the ureteroscope through the side of the retained stent is technically challenging. More invasive procedures, such as the percutaneous approach and open ureterotomy, have also been described.^19,23,25^ The telescopic access sheath method described in this report may represent a novel and minimally invasive method which was effective in all three cases without any complications.

Access sheaths of various sizes and lengths are available from various manufacturers. The selection of an access sheath depends on the size and etiology of the retained stents. These procedures should be performed under fluoroscopic guidance with extreme caution to avoid ureteral injuries. If there is buckling of the access sheath or difficulty in advancing the access sheath, the procedure should be abandoned. Excessive force should be avoided while advancing the access sheath. If a gentle and controlled attempt of the telescopic access sheath method fails to remove the retained stent, an alternative procedure should be considered.

The techniques described in this article have limitations, owing to the small size of the case series. These techniques are not suitable for patients in whom the lower end of the retained stent cannot be pulled out of the external urinary meatus. This can especially occur in male patients because of the longer length of the urethra. However, we easily pulled out the lower end of the stent from the urinary meatus in all male patients in this study. This technique may not be suitable for heavily encrusted stents. The telescopic access sheath technique should be considered experimental until its efficacy and safety are proven in a larger series.

## Conclusions

Encrustation and knot formation are known complications of retained ureteral stents. The management of such patients may be challenging and may require invasive procedures. The telescoping technique described in this report is a simple method that can be used before proceeding with a more invasive procedure. The small sample size described in this series is, however, a limitation of this study. A larger case series is required to establish the efficacy and safety of this procedure.

## Abbreviations Used

CT: computed tomography
KUB: kidneys, ureters, and bladder

## References

[1] Zimskind PD, Fetter TR, Wilkerson JL. Clinical use of long-term indwelling silicone rubber ureteral splints inserted cystoscopically. J Urol 1967;97:840–844. 6025928 10.1016/S0022-5347(17)63130-6

[2] Dyer RB, Chen MY, Zagoria RJ, Regan JD, Hood CG, Kavanagh PV. Complications of ureteral stent placement. Radiographics 2002;22:1005–1022. 12235330 10.1148/radiographics.22.5.g02se081005

[3] Baldwin DD, Juriansz GJ, Stewart S, Hadley R. Knotted ureteral stent: A minimally invasive technique for removal. J Urol 1998;159:2065–2066. 9598519 10.1016/S0022-5347(01)63248-8

[4] Small AC, Thorogood SL, Shah O, Healy KA. Emerging mobile platforms to aid in stone management. Urol Clin North Am 2019;46:287–301. 30961861 10.1016/j.ucl.2018.12.010

[5] el-Faqih SR, Shamsuddin AB, Chakrabarti A, et al. Polyurethane internal ureteral stents in treatment of stone patients: Morbidity related to indwelling times. J Urol 1991;146:1487–1491. 1942324 10.1016/s0022-5347(17)38146-6

[6] Tunney MM, Keane PF, Jones DS, Gorman SP. Comparative assessment of ureteral stent biomaterial encrustation. Biomaterials 1996;17:1541–1546. 8853126 10.1016/0142-9612(96)89780-8

[7] Ramsay JW, Crocker RP, Ball AJ, et al. Urothelial reaction to ureteric intubation. A clinical study. Br J Urol 1987;60:504–505. 3427332 10.1111/j.1464-410x.1987.tb05029.x

[8] Robert M, Boularan AM, El Sandid M, Grasset D. Double-J ureteric stent encrustations: Clinical study on crystal formation on polyurethane stents. Urol Int 1997;58:100–104. 9096271 10.1159/000282959

[9] Tomer N, Garden E, Small A, Palese M. Ureteral stent encrustation: epidemiology, pathophysiology, management and current technology. J Urol 2021;205:68–77. 32856981 10.1097/JU.0000000000001343

[10] Vanderbrink BA, Rastinehad AR, Ost MC, Smith AD. Encrusted urinary stents: Evaluation and endourologic management. J Endourol 2008;22:905–912. 18643720 10.1089/end.2006.0382

[11] Lam JS, Gupta M. Tips and tricks for the management of retained ureteral stents. J Endourol 2002;16:733–741. 12542876 10.1089/08927790260472881

[12] Pietropaolo A, Whitehurst L, Somani BK. Piecemeal retrograde removal of encrusted and encased stuck ureteral stent: Video tips and tricks. Videourology 2020;34. DOI: 10.1089/vid.2019.0057.

[13] Thomas A, Cloutier J, Villa L, Letendre J, Ploumidis A, Traxer O. Prospective analysis of a complete retrograde ureteroscopic technique with holmium laser stent cutting for management of encrusted ureteral stents. J Endourol 2017;31:476–481. 28292198 10.1089/end.2016.0816

[14] Somers WJ. Management of forgotten or retained indwelling ureteral stents. Urology 1996;47:431–435. 8633417 10.1016/S0090-4295(99)80468-3

[15] Smet G, Vandeursen H, Baert L. Extracorporeal shock wave lithotripsy for calcified ureteral catheter. Urol Int 1991;46:211–212. 2053235 10.1159/000282136

[16] Rana AM, Sabooh A. Management strategies and results for severely encrusted retained ureteral stents. J Endourol 2007;21:628–632. 17638560 10.1089/end.2006.0250

[17] Mistry K, Pal P, Chitale S. A simple two-stage “bailout” technique for the removal of an unyielding ureteric stent. Urology 2013;82:242–244. 23601439 10.1016/j.urology.2013.02.036

[18] Singh I, Gupta NP, Hemal AK, Aron M, Seth A, Dogra PN. Severely encrusted polyurethane ureteral stents: Management and analysis of potential risk factors. Urology 2001;58:526–531. 11597531 10.1016/s0090-4295(01)01317-6

[19] Kondo N, Yoshino Y, Shiono Y, Hasegawa Y. [A case demonstrating knot formation at the upper end of a ureteral stent]. Hinyokika Kiyo 2005;51:385–387. 16050477

[20] Basavaraj DR, Gill K, Biyani CS. Case report: Knotted ureteral stent in patient with ileal conduit: Conservative approach for retrieval. J Endourol 2007;21:90–93. 17263617 10.1089/end.2006.0171

[21] Picozzi S, Carmignani L. A knotted ureteral stent: A case report and review of the literature. Urol Ann 2010;2:80–82. 20882161 10.4103/0974-7796.65108 PMC2943687

[22] Tempest H, Turney B, Kumar S. Novel application of an established technique for removing a knotted ureteric stent. BMJ Case Rep 2011;2011:bcr1120103528. 10.1136/bcr.11.2010.3528 PMC4545063 22701009

[23] Bhirud P, Giridhar V, Hegde P. Midureteric knotted stent removed by percutaneous access!. Urol Ann 2012;4:106–107. 22629007 10.4103/0974-7796.95557 PMC3355692

[24] Nettle J, Huang JG, Rao R, Costello AJ. Ureteroscopic holmium laser ablation of a knotted ureteral stent. J Endourol 2012;26:968–970. 22494009 10.1089/end.2012.0081

[25] Kim MS, Lee HN, Hwang H. Knotted stents: Case report and outcome analysis. Korean J Urol 2015;56:405–408. 25964843 10.4111/kju.2015.56.5.405 PMC4426514

[26] Manohar P, Kan WT, Ranasinghe WK, Cetti RJ, McCahy P. Knotted multi-length ureteric stents: A case series. ANZ J Surg 2016;86:413–414. 24888407 10.1111/ans.12689

[27] Duty B, Okhunov Z, Okeke Z, Smith A. Medical malpractice in endourology: Analysis of closed cases from the State of New York. J Urol 2012;187:528–532. 22177166 10.1016/j.juro.2011.10.045

[28] Acosta-Miranda AM, Milner J, Turk TM. The FECal Double-J: A simplified approach in the management of encrusted and retained ureteral stents. J Endourol 2009;23:409–415. doi:10.1089/end.2008.0214 19265471

[29] Arenas JL, Shen JK, Keheila M, et al. Kidney, ureter, and bladder (KUB): A novel grading system for encrusted ureteral stents. Urology 2016;97:51–55. 27421780 10.1016/j.urology.2016.06.050

[30] Manzo BO, Alarcon P, Lozada E, et al. A novel visual grading for ureteral encrusted stent classification to help decide the endourologic treatment. J Endourol 2021;35:1314–1319. 33730863 10.1089/end.2020.1225

